# MRI-based intra-tumoral ecological diversity features and temporal characteristics for predicting microvascular invasion in hepatocellular carcinoma

**DOI:** 10.3389/fonc.2025.1510071

**Published:** 2025-03-03

**Authors:** Yuli Zeng, Huiqin Wu, Yanqiu Zhu, Chao Li, Dongyang Du, Yang Song, Sulian Su, Jie Qin, Guihua Jiang

**Affiliations:** ^1^ Department of Medical Imaging, The Affiliated Guangdong Second Provincial General Hospital of Jinan University, Guangzhou, Guangdong, China; ^2^ Department of Radiology, Third Affiliated Hospital of Sun Yat-sen University, Guangzhou, Guangdong, China; ^3^ School of Computer Science, Inner Mongolia University, Inner Mongolia, China; ^4^ Magnetic Resonance (MR) Scientific Marketing, Siemens Healthineers Ltd., Shanghai, China; ^5^ Department of Radiology, Xiamen Humanity Hospital of Fujian Medical University, Xiamen, Fujian, China; ^6^ Guangzhou Key Laboratory of Molecular Functional Imaging and Artificial Intelligence for Major Brain Diseases, Guangdong Second Provincial General Hospital, Guangzhou, Guangdong, China

**Keywords:** intra-tumoral heterogeneity, temporal features, microvascular invasion, radiomics, ensemble learning

## Abstract

**Objective:**

To investigate the predictive value of radiomics models based on intra-tumoral ecological diversity (iTED) and temporal characteristics for assessing microvascular invasion (MVI) in patients with hepatocellular carcinoma (HCC).

**Material and Methods:**

We retrospectively analyzed the data of 398 HCC patients who underwent dynamic contrast-enhanced MRI with Gd-EOB-DTPA (training set: 318; testing set: 80). The tumors were segmented into five distinct habitats using case-level clustering and a Gaussian mixture model was used to determine the optimal clusters based on the Bayesian information criterion to produce an iTED feature vector for each patient, which was used to assess intra-tumoral heterogeneity. Radiomics models were developed using iTED features from the arterial phase (AP), portal venous phase (PVP), and hepatobiliary phase (HBP), referred to as M_iTED-AP_, M_iTED-PVP_, and M_iTED-HBP_, respectively. Additionally, temporal features were derived by subtracting the PVP features from the AP features, creating a delta-radiomics model (M_Delta_). Conventional radiomics features were also extracted from the AP, PVP, and HBP images, resulting in three models: M_CVT-AP_, M_CVT-PVP_, and M_CVT-HBP_. A clinical-radiological model (CR model) was constructed, and two fusion models were generated by combining the radiomics or/and CR models using a stacking algorithm (fusion_R and fusion_CR). Model performance was evaluated using AUC, accuracy, sensitivity, and specificity.

**Results:**

The M_Delta_ model demonstrated higher sensitivity compared to the M_CVT-AP_ and M_CVT-PVP_ models. No significant differences in performance were observed across different imaging phases for either conventional radiomics (*p* = 0.096–0.420) or iTED features (*p* = 0.106–0.744). Similarly, for images from the same phase, we found no significant differences between the performance of conventional radiomics and iTED features (AP: *p* = 0.158; PVP: *p* = 0.844; HBP: *p* = 0.157). The fusion_R and fusion_CR models enhanced MVI discrimination, achieving AUCs of 0.823 (95% CI: 0.816–0.831) and 0.830 (95% CI: 0.824–0.835), respectively.

**Conclusion:**

Delta radiomics features are temporal and predictive of MVI, providing additional predictive information for MVI beyond conventional AP and PVP features. The iTED features provide an alternative perspective in interpreting tumor characteristics and hold the potential to replace conventional radiomics features to some extent for MVI prediction.

## Introduction

Hepatocellular carcinoma (HCC) is the most common form of primary liver cancer, ranking sixth in global incidence and third in mortality rate (1). Although surgical resection and liver transplantation have been shown to be effective for HCC, the high recurrence rate remains a major factor contributing to poor overall survival (2). Microvascular invasion (MVI), characterized by the presence of cancer cell clusters within endothelial-lined vascular spaces visible under microscopy (3), is an important predictor of recurrence and reduced survival in HCC patients (4, 5) and also plays a key role in determining treatment strategies (6). Currently, MVI is typically diagnosed through the examination of postoperative surgical specimens. However, needle biopsies often have low diagnostic yields and pose a risk of tumor implantation, making the preoperative and noninvasive assessment of MVI particularly challenging.

Radiomics, which provides important insights into tumor heterogeneity and the tumor microenvironment (7), offers potential for the preoperative identification of MVI. In recent years, radiomics models based on dynamic contrast-enhanced MRI have gained attention for their potential to predict MVI (8, 9). Multi-sequence and multi-parameter radiomics models have demonstrated superior predictive capabilities compared to models based on single-sequence imaging (8). Gadolinium ethoxybenzyl diethylenetriamine pentaacetic acid-enhanced MRI (Gd-EOB-DTPA MRI) is commonly used to improve the detection and characterization of HCC in clinical practice (10–12), and it has shown utility in evaluating tumor features related to MVI (13, 14).Thus, images captured during the hepatobiliary phase (HBP) are particularly valuable for defining tumor boundaries (15).

Delta radiomics involves the evaluation of relative changes in radiomic features over time (16, 17). HCC typically exhibits arterial phase (AP) hyperenhancement followed by washout in the portal venous phase (PVP). Therefore, changes in radiomic features derived from dynamic contrast-enhanced imaging can serve as important predictive biomarkers for MVI (18). Previous studies have primarily focused on individual imaging phases, often neglecting the temporal characteristics of the tumor (19, 20). In this study, we utilized deltaradiomics by subtracting PVP features from AP features to capture temporal variations that could enhance the prediction of MVI.

Previous studies mainly extracted radiomic features from the entire tumor without adequately addressing inter-tumoral heterogeneity (21). However, we believe that radiomic habitat analysis, which uses clustering methods to identify similar voxel groupings and describe environmental habitats based on ecological and biodiversity principles (22), could provide a more detailed understanding of the heterogeneous nature of HCC. Given the highly heterogeneous nature of HCC, the quantitative characterization of distinct intra-tumoral habitats may offer valuable predictive information for MVI. In this study, we applied radiomic habitat analysis on AP, PVP, and HBP images from Gd-EOB-DTPA MRI to investigate the potential of intra-tumoral habitat characteristics in predicting MVI.

## Materials and methods

### Study population

This study included 312 HCC patients from Hunan Provincial People’s Hospital/The First Affiliated Hospital of Hunan Normal University (referred to as dataset A) and 86 patients from the Third Affiliated Hospital of Sun Yat-sen University (referred to as dataset B) between February 2018 and October 2023. Institutional review board approval was obtained from each participating center. The inclusion criteria were: (1) a solitary tumor, (2) pathologically confirmed HCC following surgical resection, (3) available information on MVI status and grade, and (4) preoperative Gd-EOB-DTPA MRI conducted within two weeks prior to surgery. The exclusion criteria were: (1) macrovascular invasion, (2) prior HCC treatment before MRI (e.g., radiofrequency ablation, microwave ablation, or transcatheter arterial chemoembolization), (3) tumors larger than 10 cm in maximum diameter (as previous studies (23–25) have shown a greater likelihood of MVI in such cases), (4) inadequate MRI quality, and (5) missing pathological or clinical data. The two datasets were combined to form a total cohort of 398 patients. A randomly selected 20% of this cohort was designated as a fixed test set, and the remaining 80% of the cases were used for 5-fold cross-validation. Clinical data, including variables such as age, gender, etiology, cirrhosis, MVI status, and pathological differentiation, were extracted from electronic medical records and are summarized in **Table 1**.

**Table 1:** Clinical and radiologic information of HCC cohorts.

Characteristics	Training (N = 318)	Testing (N = 80)	P value^b^
Patient demographics			
Age (year)	56 (22–80)	55 (25–74)	0.993
Gender M F	271 47	67 13	0.878
Etiology			
HBV infection^a^	160 (50)	49 (61)	0.104
Radiological features			
Tumor size (cm)	4.24 ± 2.14	4.04 ± 2.04	0.435
Nonsmooth tumor margin (present)^a^	189 (59)	44 (55)	0.553
Enhancing capsule (present)^a^	226 (71)	52 (65)	0.357
Intertumoral artery (present)^a^	82 (26)	23 (29)	0.692
Arterial peritumoral enhancement (present)^a^	108 (34)	23 (29)	0.451
Peritumoral hypointensity on HBP (present)^a^	103 (32)	20 (25)	0.253
Pathological parameters			
Degree of differentiation^a^			0.367
well	24 (7)	4 (5)	
moderate	238 (75)	64 (80)	
poor	56 (18)	12 (15)	
Cirrhosis (stage of fibrosis 4)^a^	120 (38)	36 (45)	0.288
MVI^a^	132 (42)	33 (41)	1.000

HBV, hepatitis B virus; MVI, microvascular invasion; HBP, hepatobiliary phase.

a Data are numbers of patients, and data in parentheses are percentages.

b The *p*-value for categorical variables were calculated using the chi-square test, while those for continuous variables were calculated using the Mann-Whitney U test.

### Imaging protocol

For dataset A, MRI scans were performed using 1.5T or 3.0T MRI machines from GE (Signal Greator, Premier), Philips (Achieva, Ingenia), and Siemens (Magnetom Trio, Magnetom Prisma, Vida). For dataset B, MRI scans were conducted using 1.5T or 3.0T MRI systems from GE (Optima MR360, Signa Excite, Discovery MR750, Signa Architect), Philips (Achieva), Siemens (Magnetom Prisma), and United Imaging (uMR790). All patients underwent fat-saturated T1-weighted pre-contrast scans, followed by scans in the AP, PVP, and HBP. AP images were acquired 20-30 seconds after gadolinium contrast injection, PVP images 60-70 seconds post-injection, and HBP images were taken 20-30 minutes after contrast administration.

### Assessment of radiological features

Two experienced radiologists, each with over ten years of experience in MRI diagnostics, independently and blindly assessed the radiological features of the tumors. A consensus was reached regarding the following six characteristics: (1) tumor size, (2) non-smooth tumor margin, (3) radiological capsules (26), (4) intratumoral artery (27), (5) arterial peritumoral enhancement (28), and (6) peritumoral hypointensity on HBP (29).

### Image preprocessing and feature extraction

Primary tumors from the HBP images were manually delineated by a senior radiologist, Yuli Zeng, with over 15 years of experience, using the ITK-SNAP 3.4 software platform (www.itksnap.org). The AP and PVP images were then registered to the HBP images, which served as reference images. To correct for low-frequency intensity nonuniformity, N4 bias field correction (30) was applied to all images. All images were resampled to an isotropic voxel size of 1 × 1 × 1 mm^3^ using B-spline interpolation, while the delineated tumor masks were resampled using nearest neighbor interpolation.

For each sequence (i.e., AP, PVP, and HBP), 105 radiomics features were extracted from the original images using the PyRadiomics package (31). These features included 14 shape features and 91 texture features. Additionally, texture features were extracted using wavelet filters (HHH, HHL, HLH, HLL, LHH, LHL, LLH, LLL) and Laplacian of Gaussian filters with sigmas of 2.0, 3.0, 4.0 and 5.0, which resulted in a comprehensive set of 1197 features for each sequence.

### Delta radiomics features

The images of HCC patients typically exhibit AP hyperenhancement followed by washout in the PVP (16). To assess radiomic changes during dynamic contrast enhancement, the features from the AP images were compared to those from the PVP images using the following Equation 1:


(1) $$F_{Delta}=F_{AP}-F_{PVP}$$

Where $F_{Delta}$ represents the change in features between AP images and PVP images, which is time-related and predictive to MVI, $F_{AP}$ represents the features extracted from AP images, and $F_{PVP}$ refers to the features generated from the PVP images.

### Intra-tumoral subregion partitioning and ecological diversity feature generation

Radiomics offers detailed insights into tumor phenotypes and the tumor microenvironment (32). To capture intra-tumoral heterogeneity, we performed intra-tumoral habitat partitioning in two steps: case-based clustering and subregion feature extraction. Case-based clustering was conducted independently for each tumor using the k-means algorithm with squared Euclidean distances between voxel intensities. The number of clusters was set to five due to the small tumor volumes in this study. The clustering process is performed using the in-house nnFAE software. In the subregion feature extraction step, radiomics features, including histogram, GLCM, GLRLM, NGTDM, GLSZM and GLDM, were extracted from each subregion without applying additional filters.

Subsequently, we applied a Gaussian mixture model to perform unsupervised clustering of radiomic features across all tumor habitats. The optimal number of clusters, representing the diversity of the tumor ecosystem, was determined using the Bayesian Information Criterion (BIC) (33), which generated an intra-tumoral ecological diversity (iTED) feature vector for each patient, which could then be used for further analysis. Each iTED feature reflects the optimal number of clusters corresponding to specific radiomic features. For example, the iTED_entropy feature represents the optimal number of clusters for assessing tumor heterogeneity, using traditional entropy as a metric. While conventional entropy measures the unpredictability or variability of image values, iTED_entropy quantifies the complexity of intra-tumoral heterogeneity by evaluating entropy at the cluster level. This iTED feature vector provides a novel approach to tumor characterization, potentially offering new insights into tumor behavior and structure (34).

**Figure 1:**
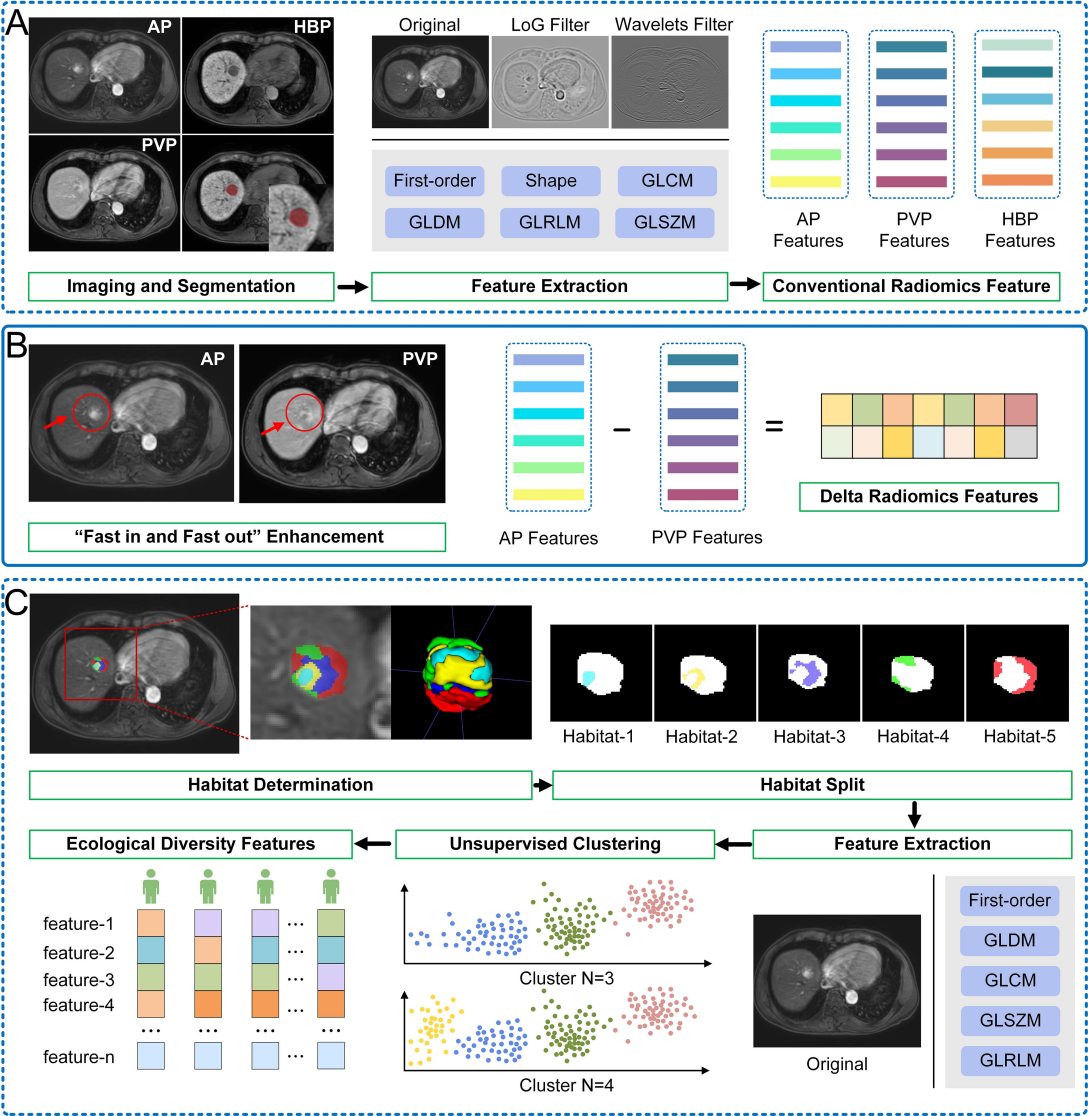
Schematic shows the workflow of the generation of conventional radiomics features, delta radiomics features and ecological diversity features. **(A)** Conventional radiomics features. **(B)** Delta radiomics features. **(C)** Ecological diversity features.

The generation of the conventional radiomics features, delta radiomics features and iTED features is shown in **Figure 1**. To address potential variability in the radiomics features caused by differences in imaging protocols across the two centers, the ComBat harmonization method (35) was applied.

### Feature stability assessment

To preselect features with high stability, we simulated delineation perturbations based on the training cohort. Morphological operations, including dilation and erosion, were applied slice-by-slice using a circular structural element with distances of 1 mm and 2 mm. This process generated four distinct VOIs, labeled D1, D2, E1, and E2. To assess feature stability, we used the inter-class correlation coefficient (ICC) (36), classifying features as having high (ICC ≥ 0.75), moderate (0.75 > ICC ≥ 0.50), or low (ICC < 0.50) stability. Following established guidelines (37), we applied the ICC (2,1) model as defined by Shrout and Fleiss (38) and calculated the ICC using the Pingouin statistical library (https://github.com/raphaelvallat/pingouin).

ICC was calculated for all five ROIs, including the original ROI, and the dilated (1 mm and 2 mm) and eroded (1 mm and 2 mm) ROIs. Only first-order and textural features were evaluated for stability, with features having an ICC greater than 0.75 selected for the next stage of the feature selection pipeline. Shape-related features, however, were directly retained and included in the pipeline without undergoing stability evaluation.

### Feature selection

For both conventional radiomic features and delta radiomics features, the selection process began with retaining features that showed significant differences between patients with and without MVI, as determined by the Mann–Whitney U-test. Next, we selected features that achieved an Area Under the Curve (AUC) greater than 0.60 in univariate logistic regression analysis. To further refine the feature set, the minimum redundancy and maximum relevancy (mRMR) method was applied to eliminate redundant and irrelevant features. Finally, the Least Absolute Shrinkage and Selection Operator (LASSO) algorithm was used to reduce the feature set to only the most predictive features. For conventional radiomic features, this process resulted in the selection of 18 features from AP images, 13 from PVP images, and 10 from HBP images. For delta radiomics features, 14 features were retained for model development.

For the iTED features, we first applied z-score normalization to standardize the features and removed those with minimal variance. Next, features with an AUC greater than 0.55 in univariate logistic regression were retained. The LASSO algorithm was then applied, leaving 4 features from AP images, 4 from PVP images, and 9 from HBP images for further analysis.

The details of the selected features and their corresponding ICC values are provided in **Supplementary Tables S1**-**S7** of the **Supplementary Material**.

### Prediction model construction and statistical analysis

Differences in clinical and radiological characteristics between the training and test cohorts were assessed using the Mann-Whitney U test for continuous variables and the chi-square test for categorical variables. In the training dataset, five-fold cross-validation with stratified sampling was performed to ensure consistent category proportions. A random forest (RF) model was constructed to classify patients with or without MVI, and Bayesian optimization (39) was applied to fine-tune the model’s hyperparameters.

**Figure 2:**
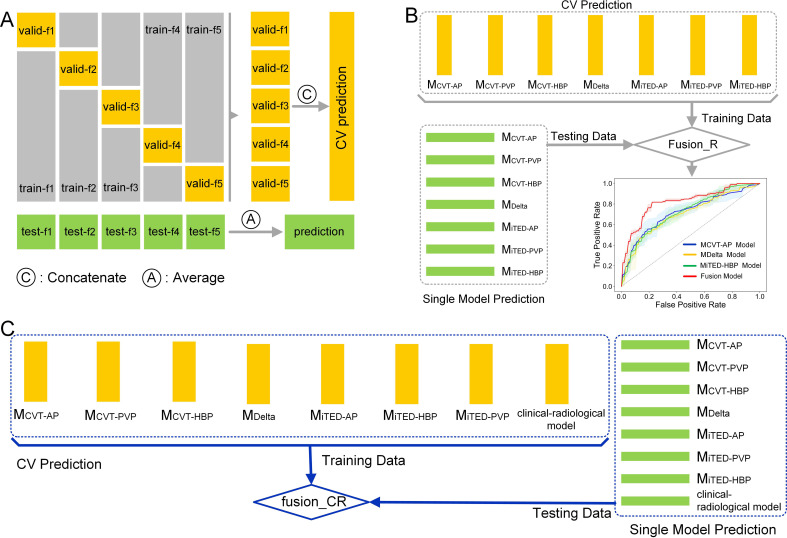
The workflow of developing the fusion model. **(A)** Cross-validation predictions and test set predictions generated by the based learner. **(B)** The training process of fusion_R model. **(C)** The training process of fusion_CR model.

Ultimately, eight model types were constructed using (1) A clinical-radiological model (CR model) using demographic, pathological, and radiological features, (2) conventional radiomic features from AP images (M_CVT-AP_), (3) conventional radiomic features from PVP images (M_CVT-PVP_), (4) conventional radiomic features from HBP images (M_CVT-HBP_), (5) delta radiomics features (M_Delta_), (6) iTED features from AP images (M_iTED-AP_), (7) iTED features from PVP images (M_iTED-PVP_), and (8) iTED features from HBP images (M_iTED-HBP_). Then, we developed a fusion_R model to combine the predictions from the above seven radiomics models using a stacking algorithm (40). Furthermore, we constructed a fusion_CR model, which combines the radiomics models with the CR model. The workflow of developing the fusion model by stacking algorithm is shown in **Figure 2**.

The performance of the models in predicting MVI was evaluated using the Area Under the Receiver Operating Characteristic Curve (AUC) with 95% confidence intervals, as well as accuracy (ACC), sensitivity, and specificity. Delong’s test was employed to compare the AUCs of different models, with statistical significance set at *p* < 0.05.

## Results

### Performance of the CR model

A total of 398 HCC patients were included in the study, with 318 patients in the training dataset (mean age 56 years; 271 males, 47 females) and 80 patients in the testing dataset (mean age 55 years; 67 males, 13 females). As shown in **Table 1**, the two sets were well-balanced as there were no statistically significant differences in clinical-radiological characteristics either between the training and testing sets or within each set (*p* = 0.104-1.000).

Univariate analysis identified one demographic factor (HBV infection), four radiological factors (tumor size, nonsmooth tumor margin, arterial peritumoral enhancement, and peritumoral hypointensity on HBP), and two pathological factors (degree of differentiation and cirrhosis, stage 4 fibrosis) as being associated with MVI in the training set. Multivariable analysis (**Table 2**) revealed that tumor size (OR = 1.20, 95% CI: 1.06–1.36, *p* < 0.001), nonsmooth tumor margin (OR = 2.84, 95% CI: 1.63–5.06, *p* < 0.001), and cirrhosis (stage 4 fibrosis) (OR = 2.02, 95% CI: 1.19–3.50, *p* = 0.01) were significant predictors of MVI and were incorporated into the clinical-radiological (CR) model. The CR model achieved an AUC of 0.784 (95% CI: 0.766–0.802) in the training dataset, 0.722 (95% CI: 0.661–0.784) in the validation dataset, and 0.677 (95% CI: 0.610–0.744) in the testing dataset.

**Table 2:** Stepwise multivariable logistic regression analysis for clinical and radiological variables.

Variable	*β*	OR (95% CI)	P value
HBV infection	0.41	1.51 (0.83-2.78)	0.18
Tumor size	0.18	1.20 (1.06-1.36)	< 0.001*
Nonsmooth tumor margin	1.05	2.84 (1.63-5.06)	< 0.001*
Arterial peritumoral enhancement	-0.04	0.96 (0.38-2.34)	0.93
Peritumoral hypointensity on HBP	0.73	2.08 (0.85-5.22)	0.11
Degree of differentiation	-0.04	0.96 (0.74-1.18)	0.67
Cirrhosis (stage of fibrosis 4)	0.71	2.02 (1.19-3.50)	0.01*

These analyses were performed using the training data set (n = 318).

CI, confidence interval; OR, odds ratio; HBV, hepatitis B virus; HBP, hepatobiliary phase. *The P value is statistically significant.

### Performance of models based on conventional radiomics features

**Figure 3:**
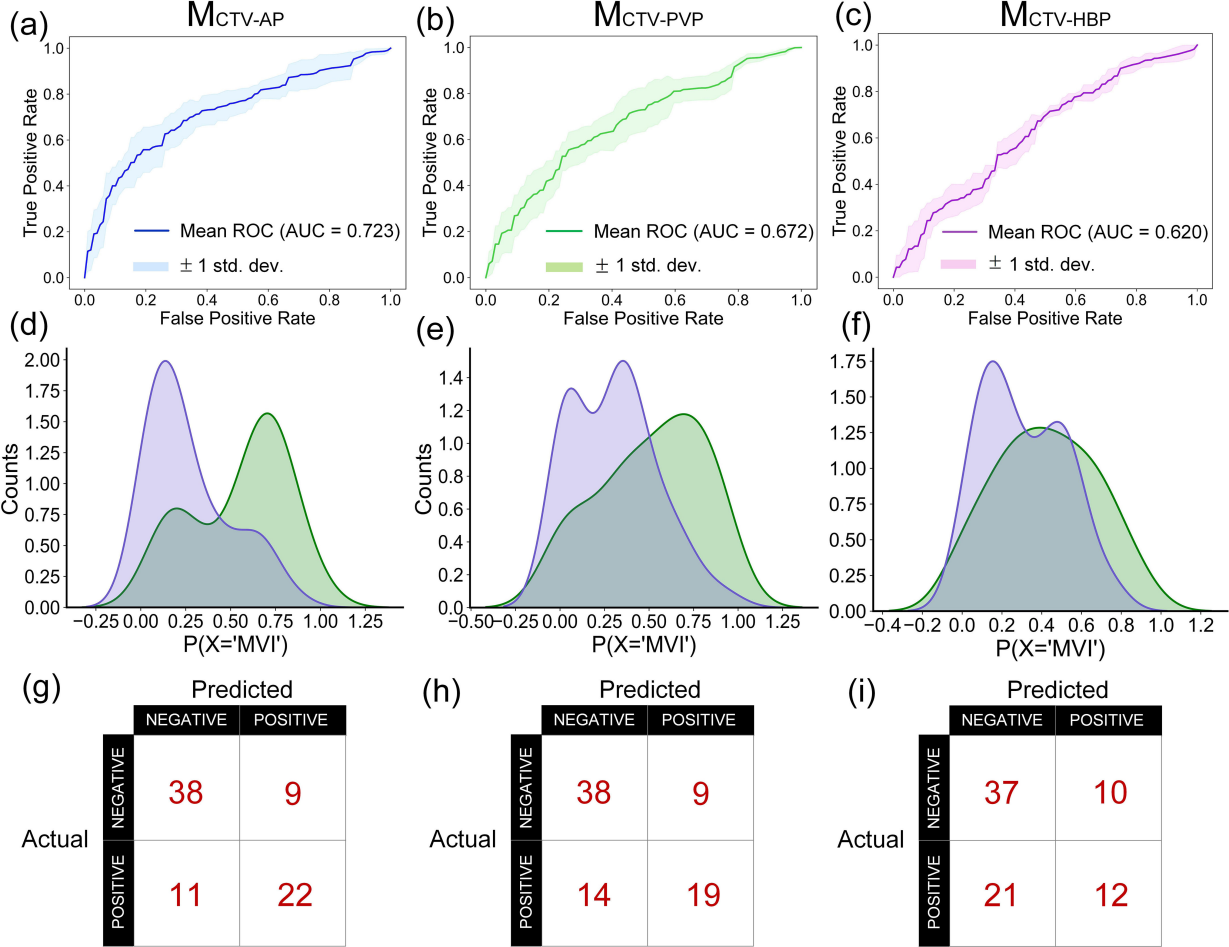
The mean ROC curves, the probability distribution of classes and the confusion matrixes of M_CVT-AP_, M_CVT-PVP,_ and M_CVT-HBP_ model. **(a–c)**, the mean ROC curves of M_CVT-AP_ **(a)**, M_CVT-PVP_ **(b)**, and M_CVT-HBP_ **(c)** model, where the shaded region indicates 95% confidence intervals. **(d–f)** depict the probability distribution of classes for the M_CVT-AP_ **(d)**, M_CVT-PVP_ **(e)**, and M_CVT-HBP_ **(f)** model. Presented here is the model from one of the folds that is closest to the cross-validation mean. **(g-i)** depict the confusion matrixes for the M_CVT-AP_ **(g)**, M_CVT-PVP_ **(h)**, and M_CVT-HBP_ **(i)** model. Presented here is the model from one of the folds that is closest to the cross-validation mean.

The performance of models based on conventional radiomics features is presented in **Table 3**. The M_CVT-AP_ model achieved an AUC of 0.723 (95% CI: 0.655–0.789) in the testing cohort, demonstrating superior diagnostic performance compared to the M_CVT-PVP_ model (AUC = 0.672, 95% CI: 0.611–0.734) and the M_CVT-HBP_ model (AUC = 0.620, 95% CI: 0.601–0.639). The higher signal contrast within and between tumors on AP images, due to significant enhancement, likely contributed to this improved performance. Conversely, the M_CVT-HBP_ model exhibited lower sensitivity (0.496), likely due to the minimal signal variation observed in HCC lesions during the HBP phase. **Figure 3** illustrates the mean receiver operating characteristic (ROC) curves, the probability distribution of classes, and the confusion matrices for the M_CVT-AP_, M_CVT-PVP_, and M_CVT-HBP_ models.

**Table 3:** The performance of models based on conventional radiomics features.

Model		AUC	ACC	Sensitivity	Specificity
M_CVT-AP_	Training	0.884 (0.865, 0.903)	0.813 (0.781, 0.845)	0.806 (0.714, 0.899)	0.817 (0.723, 0.911)
M_CVT-PVP_		0.870 (0.829, 0.910)	0.806 (0.754, 0.857)	0.795 (0.733, 0.858)	0.813 (0.709, 0.917)
M_CVT-HBP_		0.885 (0.865, 0.905)	0.810 (0.770, 0.849)	0.830 (0.751, 0.907)	0.796 (0.691, 0.900)
M_CVT-AP_	Validation	0.766 (0.705, 0.826)	0.719 (0.637, 0.802)	0.689 (0.530, 0.848)	0.741 (0.600, 0.881)
M_CVT-PVP_		0.733 (0.660, 0.806)	0.707 (0.634, 0.780)	0.644 (0.447, 0.841)	0.752 (0.638, 0.867)
M_CVT-HBP_		0.749 (0.701, 0.797)	0.675 (0.610, 0.741)	0.688 (0.557, 0.820)	0.665 (0.518, 0.812)
M_CVT-AP_	Testing	0.723 (0.655, 0.789)	0.692 (0.629, 0.755)	0.635 (0.508, 0.763)	0.731 (0.612, 0.850)
M_CVT-PVP_		0.672 (0.611, 0.734)	0.655 (0.622, 0.687)	0.520 (0.448, 0.593)	0.748 (0.691, 0.805)
M_CVT-HBP_		0.620 (0.601, 0.639)	0.595 (0.542, 0.647)	0.496 (0.382, 0.611)	0.663 (0.524, 0.802)

The results were reported as the mean of cross-validation with a 95% confidence interval (CI).

AUC, area under ROC curve; ACC, Accuracy.

### Performance of models based on delta radiomics features

The performance of the M_Delta_ model is summarized in **Table 4**. The M_Delta_ model achieved an AUC of 0.707 (95% CI: 0.678–0.735), outperforming the M_CVT-PVP_ model (AUC = 0.672, 95% CI: 0.611–0.734) but falling short of the M_CVT-AP_ model (AUC = 0.723, 95% CI: 0.655–0.789). Importantly, the M_Delta_ model exhibited a higher sensitivity (0.672, 95% CI: 0.590–0.753) compared to the M_CVT-AP_ model (0.635, 95% CI: 0.508–0.763) and the M_CVT-PVP_ model (0.520, 95% CI: 0.448–0.593), suggesting that delta radiomics features, which capture time-related changes, provide valuable predictive information for MVI beyond what is offered by AP and PVP features alone.

**Table 4:** The performance of models based on delta radiomics features.

	AUC	ACC	Sensitivity	Specificity
Training	0.923 (0.911, 0.935)	0.837 (0.825, 0.849)	0.896 (0.843, 0.948)	0.795 (0.742, 0.849)
Validation	0.840 (0.688, 0.875)	0.723 (0.602, 0.843)	0.718 (0.605, 0.831)	0.724 (0.465, 0.982)
Testing	0.707 (0.678, 0.735)	0.650 (0.615, 0.684)	0.672 (0.590, 0.753)	0.633 (0.547, 0.720)

The results were reported as the mean of cross-validation with a 95% confidence interval (CI).

AUC, area under ROC curve; ACC, Accuracy.

### Performance of models based on iTED radiomics features

**Figure 4:**
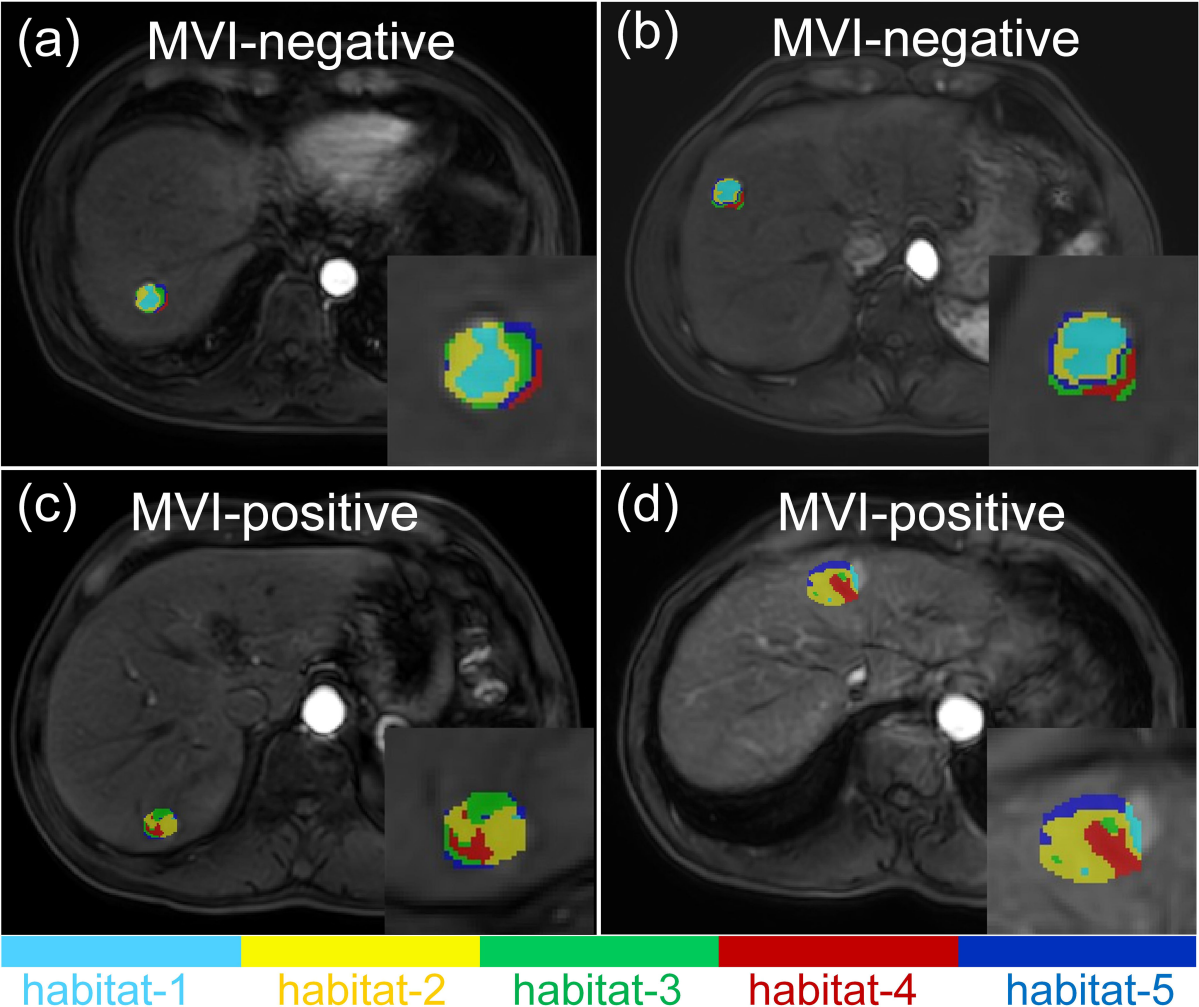
Representative MRI images of two MVI-negative **(a, b)** as well as two MVI-positive HCCs **(c, d)**.

**Figure 5:**
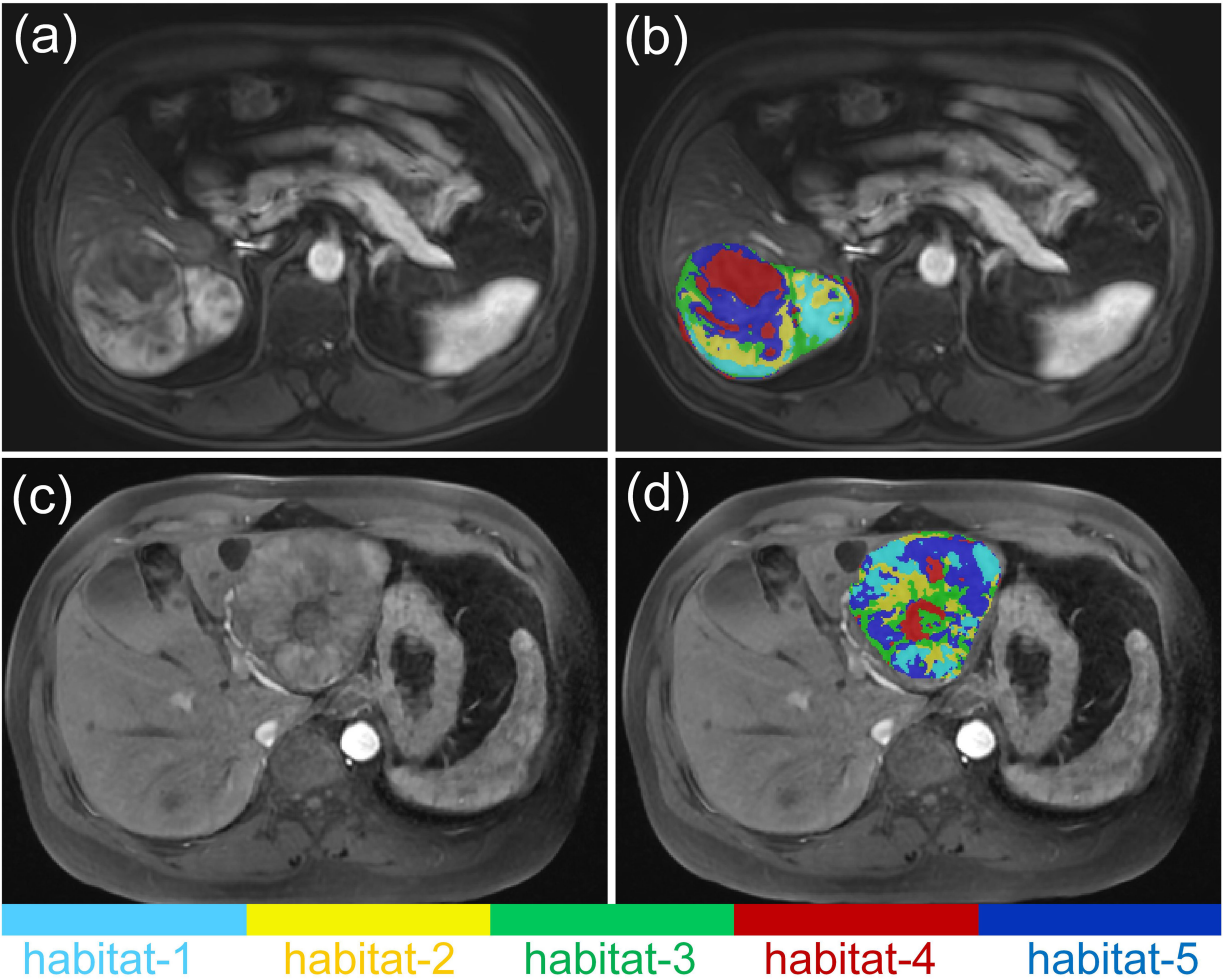
Spatial habitats clustered by similar voxels using AP images are demonstrated for a 68-year-old male patient **(a, b)** and a 51-year-old male patient **(c, d)**. The habitats are defined as follows: habitat-1, regions with high enhancement; habitat-2, regions with medium to medium-high enhancement; habitat-3, regions with low or no enhancement; habitat-4, regions of cystic degeneration and necrosis; habitat-5, regions with medium-low enhancement.

As shown in **Table 5**, the M_iTED-HBP_ model (AUC = 0.727, 95% CI: 0.706–0.749) outperformed the M_iTED-AP_ model (AUC = 0.613, 95% CI: 0.575–0.651) and the M_iTED-PVP_ model (AUC = 0.691, 95% CI: 0.676–0.707). Interestingly, the performance of the iTED models in different phases was the opposite of that observed in the conventional radiomics models. However, the sensitivity of the iTED models was relatively low (Sensitivity = 0.460–0.545) in the testing cohort. Representative MRI images are displayed in **Figure 4**. MVI-positive HCC cases were found to have a higher proportion of habitat-4 in the tumor center. **Figure 5** provides a visual assessment of clustering effectiveness while emphasizing intra-tumor heterogeneity. In the figure, habitat-1 represents regions with high enhancement; habitat-2 corresponds to areas with medium to medium-high enhancement; habitat-3 includes regions with low or no enhancement; habitat-4 highlights areas of cystic degeneration and necrosis; and habitat-5 encompasses regions with medium-low enhancement.

**Table 5:** The performance of models based on iTED features.

Model		AUC	ACC	Sensitivity	Specificity
M_iTED-AP_	Training	0.699 (0.668, 0.730)	0.644 (0.618, 0.670)	0.649 (0.624, 0.675)	0.640 (0.593, 0.686)
M_iTED-PVP_		0.822 (0.804, 0.840)	0.740 (0.719, 0.762)	0.820 (0.772, 0.867)	0.684 (0.615, 0.753)
M_iTED-HBP_		0.883 (0.868, 0.897)	0.800 (0.781, 0.819)	0.818 (0.738, 0.898)	0.787 (0.721, 0.854)
M_iTED-AP_	Validation	0.639 (0.536, 0.742)	0.600 (0.500, 0.700)	0.606 (0.507, 0.705)	0.596 (0.460, 0.731)
M_iTED-PVP_		0.719 (0.645, 0.793)	0.632 (0.578, 0.686)	0.696 (0.640, 0.752)	0.585 (0.497, 0.673)
M_iTED-HBP_		0.781 (0.740, 0.824)	0.707 (0.633, 0.781)	0.734 (0.670, 0.798)	0.687 (0.589, 0.785)
M_iTED-AP_	Testing	0.613 (0.575, 0.651)	0.587 (0.558, 0.616)	0.460 (0.363, 0.557)	0.676 (0.637, 0.715)
M_iTED-PVP_		0.691 (0.676, 0.707)	0.677 (0.657, 0.697)	0.545 (0.429, 0.660)	0.769 (0.677, 0.862)
M_iTED-HBP_		0.727 (0.706, 0.749)	0.690 (0.673, 0.707)	0.496 (0.463, 0.530)	0.825 (0.786, 0.864)

The results were reported as the mean of cross-validation with a 95% confidence interval (CI).

AUC, area under ROC curve; ACC, Accuracy.

### Performance of the fusion model

**Figure 6:**
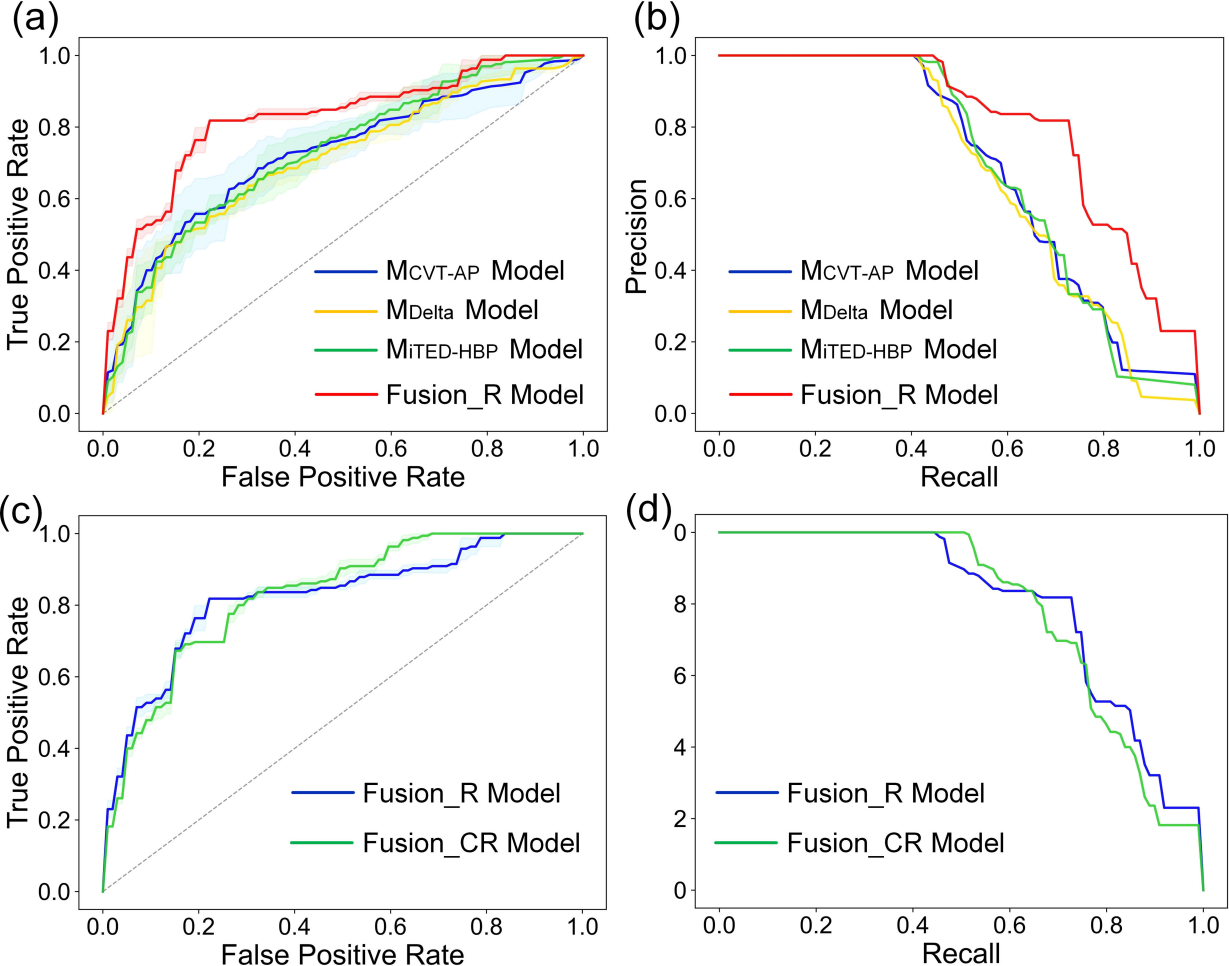
The receiver operating characteristic curves (ROC curves) of **(a)** M_CVT-AP_ model, M_Delta_ model, M_iTED-HBP_ model and the fusion_R model, **(c)** the fusion_R and fusion_CR model. The precision–recall curves (PR curves) of **(b)** M_CVT-AP_ model, M_Delta_ model, M_iTED-HBP_ model and the fusion_R model, **(d)** the fusion_R and fusion_CR model.

As shown in **Table 6**, the fusion_R model demonstrated excellent discriminatory performance, achieving an AUC of 0.823 (95% CI: 0.816–0.831) and an accuracy of 0.775 (95% CI: 0.753–0.796) in the testing cohort. The fusion_R model outperformed the base classifiers (M_CVT-AP_, M_CVT-PVP_, M_CVT-HBP_, M_Delta_, M_iTED-AP_, M_iTED-PVP_, and M_iTED-HBP_) across nearly all evaluation metrics in both the validation and testing cohorts. **Figures 6a, b** shows the ROC and precision-recall (PR) curves for the fusion_R model alongside the best-performing conventional radiomics model (M_CVT-AP_), delta radiomics model (M_Delta_), and iTED model (M_iTED-HBP_).

**Table 6:** The performance of fusion_R and fusion_CR model.

	Training		Validation		Testing	
	fusion_R	Fusion_CR	fusion_R	Fusion_CR	fusion_R	Fusion_CR
AUC	0.876 (0.857, 0.896)	0.868 (0.851, 0.885)	0.869 (0.797, 0.942)	0.863 (0.800, 0.926)	0.823 (0.816, 0.831)	0.830 (0.824, 0.835)
ACC	0.799 (0.765, 0.834)	0.812 (0.786, 0.835)	0.801 (0.692, 0.910)	0.814 (0.737, 0.891)	0.775 (0.753, 0.796)	0.779 (0.772, 0.785)
Sensitivity	0.759 (0.716, 0.802)	0.734 (0.701, 0.768)	0.747 (0.564, 0.929)	0.732 (0.594, 0.870)	0.684 (0.627, 0.741)	0.672 (0.655, 0.689)
Specificity	0.834 (0.821, 0.848)	0.867 (0.847, 0.887)	0.838 (0.754, 0.922)	0.870 (0.760, 0.979)	0.816 (0.750, 0.882)	0.846 (0.834, 0.858)

The results were reported as the mean of cross-validation with a 95% confidence interval (CI).

AUC, area under ROC curve; ACC, Accuracy.

The performance of the fusion_R model was comparable to that of the fusion_CR model (AUC = 0.823 vs. AUC = 0.830, *p* = 0.718), suggesting that while clinical-radiological features had predictive value, their contribution to enhancing the radiomics-based prediction was minimal. The ROC and PR curves for the fusion_R and fusion_CR models are displayed in **Figures 6c, d**. Additionally, we applied sigmoid calibration to the fusion_R and fusion_CR models. However, the calibration resulted in no significant improvement in performance, with the fusion_R model showing a slight change (pre: 0.823 vs. post: 0.825) and the fusion_CR model exhibiting minimal variation (pre: 0.830 vs. post: 0.828). **Supplementary Figure S1** in the **Supplementary Material** presents the model calibration curves both before and after calibration.

### Comparison of performance between different models

**Figure 7:**
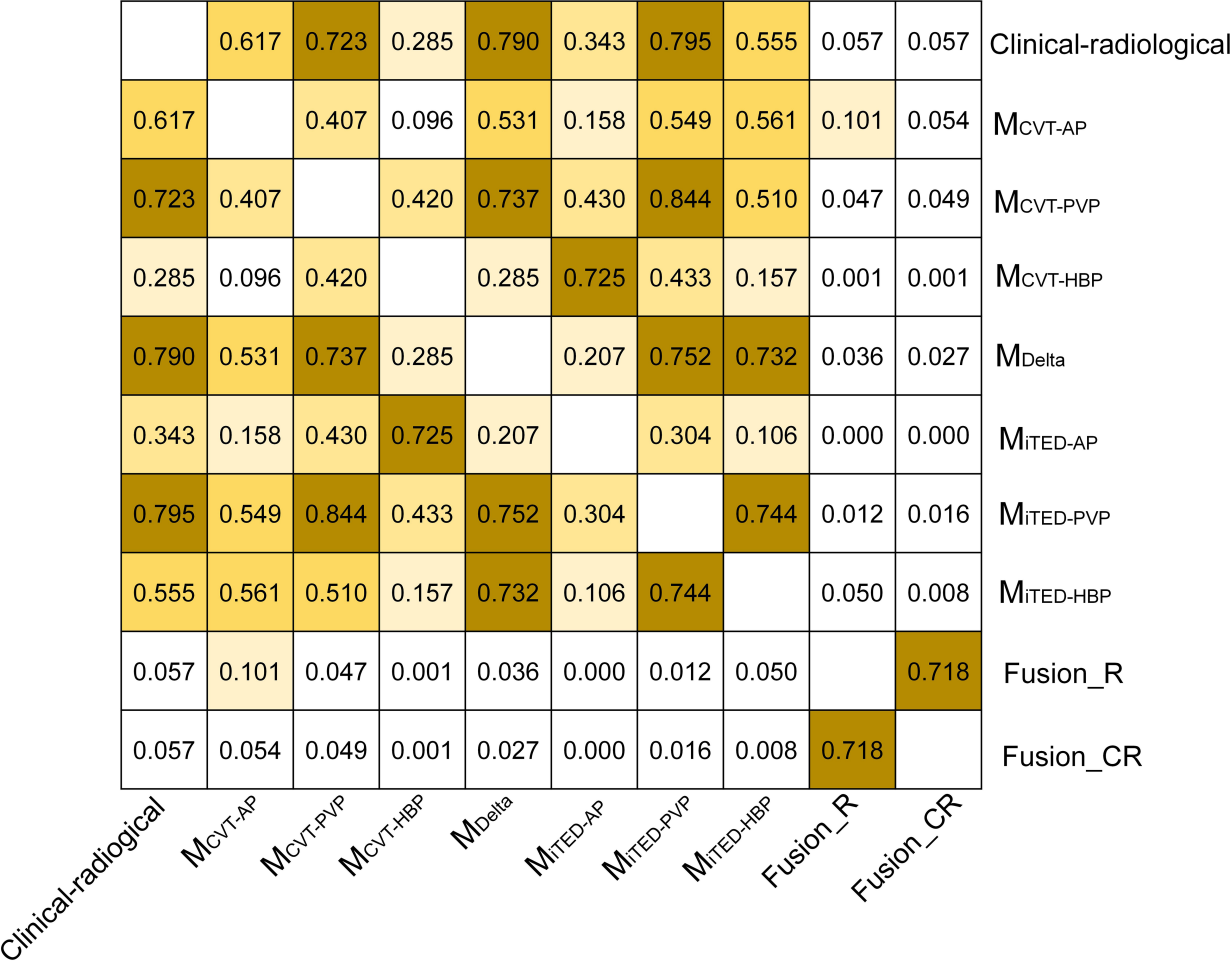
The *p*-value of the Delong test between the models.

The *p* values of the Delong test between the different models are shown in **Figure 7**. The fusion model significantly improved MVI discrimination compared to every other model (*p* = 0.000–0.050) except the M_CVT-AP_ model (fusion_R: *p* = 0.101, fusion_CR: *p* = 0.054). No significant differences were found in the performance of images from different phases, whether using conventional radiomics features (*p* = 0.096–0.420) or iTED features (*p* = 0.106–0.744). Additionally, for images from the same phase, there was no significant difference in performance between conventional radiomics features and iTED features (*p* = 0.158 for AP images, *p* = 0.844 for PVP images, and *p* = 0.157 for HBP images). These findings suggest that, although the predictive power of iTED features is not as strong as conventional radiomics features, iTED features have the potential to replace conventional radiomics features to some extent.

## Discussion

In this study, we developed and validated seven radiomics models (M_CVT-AP_, M_CVT-PVP_, M_CVT-HBP_, M_Delta_, M_iTED-AP_, M_iTED-PVP_, and M_iTED-HBP_) as well as a CR model. Additionally, two fusion models were constructed by combining radiomics models and/or the CR model using a stacking algorithm. Our findings demonstrated that both iTED features and temporal delta radiomics features exhibit substantial predictive power for MVI in HCC.

Gd-EOB-DTPA-enhanced MRI is highly effective in detecting and characterizing focal liver lesions, particularly small-size HCC. After the uptake of Gd-EOB-DTPA contrast, normal functioning hepatocytes exhibit significant enhancement, resulting in high signal intensity during the HBP. In contrast, lesions with impaired or absent hepatocyte function show varying degrees of reduced signal intensity. This marked difference in signal between tumor tissues and the surrounding liver parenchyma is more pronounced in HBP images compared to conventional contrast agents, making tumor boundaries easier to delineate (15). Additionally, key radiological features associated with MVI, such as tumor margin, capsule formation, tumor size, and peritumoral hypointensity, are more clearly visualized in HBP images (41). Our findings align with a previous study, which reported AUC and accuracy values of 0.62 and 0.64, respectively, for an HBP-based model (42).

Delta radiomics analyzes variations in imaging features at different time points, typically before and after treatment. This approach allows for the assessment of changes in features following specific steps in the patient’s care process, such as after therapy, at a particular time point, or in response to a biological event (43). HCC is predominantly supplied by the hepatic artery, which leads to distinct enhancement patterns and signal variations, particularly during the AP and PVP. These dynamic changes in imaging features can serve as strong predictors of MVI, offering additional insights beyond the static AP and PVP features. Xia et al. (18) applied delta radiomics to predict MVI using CT images, yielding AUC values of 0.76 for the internal test set and 0.72 for the external test set. Our experimental results align closely with these findings.

Recent studies have shown that tumors consist of multiple subregions or habitats, each representing clusters of tissue with similar structural, metabolic, or functional characteristics (44). In our study, we accounted for this spatial heterogeneity by dividing tumors into five habitats and extracting radiomic features from each habitat independently. The iTED feature vectors quantified intra-tumoral heterogeneity by determining the optimal number of clusters for each feature. Research has highlighted the importance of radiomic habitat analysis in evaluating MVI. For example, Zhang et al. (44) demonstrated that habitat-imaging-derived quantitative metrics from AP images were significantly associated with MVI, and a nomogram incorporating habitat-derived metrics and tumor size effectively identified MVI-positive HCC. Liu et al. (22) combined habitat modeling with a deep-learning approach based on AP, PVP, and delayed phase images to predict MVI, achieving AUC values of 0.90 in the training set and 0.86 in the validation set. Although their results slightly outperform ours (training: AUC = 0.90 vs. 0.88; validation: AUC = 0.86 vs. 0.78), our findings remain consistent with the predictive power of habitat-based radiomics.

This study demonstrated that the two fusion models significantly improved the accuracy of MVI prediction. Each of the individual models performed exceptionally well in different aspects. The M_CVT-AP_ model, in particular, had a higher AUC value in the testing cohort, indicating stronger predictive power. However, the M_iTED_ models displayed lower sensitivity, which suggests a higher risk of missing MVI-positive patients. In contrast, the M_Delta_ model exhibited notable sensitivity, making it more reliable in detecting MVI-positive cases. The fusion models outperformed all other models, and the reasons for this superior performance are twofold. First, the models were developed using images from different contrast enhancement phases, with each phase providing distinct yet complementary information. Second, the use of a stacking algorithm to combine the radiomics models and/or the CR model further enhanced predictive accuracy, reduced the risk of overfitting, and minimized assumptions related to model parameters (40).

Despite these promising findings, several limitations should be acknowledged. First, being a retrospective study, this research inherently introduced certain biases, such as variations in image acquisition times during dynamic enhanced scanning and differences in imaging parameters. Second, despite being manually delineated by an experienced associate chief diagnostic physician, ROI boundaries may still be inaccurate in cases of incomplete capsules or unclear lesion edges due to subjective interpretation. To account for inter-observer variability, we simulated variability using morphological operations (dilation and erosion), though these methods are limited in capturing actual delineation discrepancies. Third, the fusion models were created by combining all radiomics models, but alternative model combinations might exist that could further enhance the models’ performance and robustness.

In conclusion, iTED features reflecting intratumoral heterogeneity and time-related delta features demonstrated strong predictive capabilities for the preoperative and non-invasive prediction of MVI. The fusion_R and fusion_CR models provided complementary strengths and exhibited superior efficacy, offering valuable assistance in personalized clinical decision-making and improving the prognosis of HCC patients.

## Data Availability

The original contributions presented in the study are included in the article/**Supplementary Material**. Further inquiries can be directed to the corresponding authors.
