# Supplementary material for: MRI-based intra-tumoral ecological diversity features and temporal characteristics for predicting microvascular invasion in hepatocellular carcinoma
Source: Front Oncol. 2025 Mar 3;15:1510071. doi: 10.3389/fonc.2025.1510071 (PMC11911209; doi:10.3389/fonc.2025.1510071)
Supplement: Supplementary file 1 [file DataSheet1.docx]

**Supplementary Material**

***MRI-based Intra-tumoral*** ***Ecological Diversity Features and Temporal Characteristics for Predicting Microvascular Invasion in Hepatocellular Carcinoma***

Table S1. The selected features and corresponding ICC values of M_CVT-AP_ model.

| Feature | ICC |
| --- | --- |
| log-sigma-5-0-mm-3D_glcm_InverseVariance | 0.84 |
| wavelet-HHH_firstorder_Mean | 0.77 |
| wavelet-HLH_glszm_SmallAreaEmphasis | 0.81 |
| wavelet-HHL_glrlm_LongRunHighGrayLevelEmphasis | 0.93 |
| original_shape_Maximum3DDiameter | -- |
| log-sigma-4-0-mm-3D_glcm_InverseVariance | 0.80 |
| wavelet-HLL_glrlm_ShortRunLowGrayLevelEmphasis | 0.90 |
| wavelet-HLH_glszm_LowGrayLevelZoneEmphasis | 0.90 |
| wavelet-HLH_firstorder_Kurtosis | 0.76 |
| wavelet-LHH_glszm_SmallAreaEmphasis | 0.77 |
| wavelet-HLL_glszm_LowGrayLevelZoneEmphasis | 0.81 |
| wavelet-HHL_firstorder_Mean | 0.89 |
| wavelet-HHL_glszm_LargeAreaHighGrayLevelEmphasis | 0.94 |
| wavelet-HHH_glszm_SmallAreaEmphasis | 0.82 |
| original_shape_Sphericity | -- |
| wavelet-HHL_glrlm_RunEntropy | 0.91 |
| wavelet-HHL_gldm_SmallDependenceLowGrayLevelEmphasis | 0.86 |
| wavelet-HLL_glszm_SmallAreaLowGrayLevelEmphasis | 0.84 |

Table S2. The selected features and corresponding ICC values of M_CVT-PVP_ model.

| Feature | ICC |
| --- | --- |
| log-sigma-5-0-mm-3D_firstorder_90Percentile | 0.91 |
| wavelet-HLH_firstorder_Kurtosis | 0.90 |
| original_shape_Maximum3DDiameter | -- |
| wavelet-HHL_glszm_LowGrayLevelZoneEmphasis | 0.86 |
| wavelet-HHH_gldm_SmallDependenceLowGrayLevelEmphasis | 0.76 |
| wavelet-LHL_glcm_Idmn | 0.77 |
| wavelet-HLL_gldm_DependenceEntropy | 0.92 |
| wavelet-LHL_glcm_Idn | 0.82 |
| wavelet-HLL_glszm_SmallAreaLowGrayLevelEmphasis | 0.89 |
| original_shape_Sphericity | -- |
| wavelet-HHL_glszm_SmallAreaLowGrayLevelEmphasis | 0.76 |
| wavelet-LLL_glcm_JointEnergy | 0.86 |
| wavelet-LHL_glszm_SmallAreaLowGrayLevelEmphasis | 0.85 |

Table S3. The selected features and corresponding ICC values of M_CVT-HBP_ model.

| Feature | ICC |
| --- | --- |
| wavelet-HLH_glszm_SmallAreaEmphasis | 0.83 |
| wavelet-HLH_glszm_GrayLevelNonUniformity | 0.91 |
| log-sigma-2-0-mm-3D_gldm_DependenceEntropy | 0.89 |
| original_shape_Maximum3DDiameter | -- |
| wavelet-LHL_glszm_SmallAreaLowGrayLevelEmphasis | 0.78 |
| log-sigma-3-0-mm-3D_glszm_ZoneEntropy | 0.78 |
| original_shape_Maximum2DDiameterColumn | -- |
| original_shape_Sphericit | -- |
| log-sigma-3-0-mm-3D_glszm_SmallAreaLowGrayLevelEmphasis | 0.89 |
| wavelet-LLH_glrlm_RunLengthNonUniformity | 0.85 |

Table S4. The selected features and corresponding ICC values of M_Delta_ model.

| Feature | ICC |
| --- | --- |
| log-sigma-5-0-mm-3D_firstorder_Energy | 0.88 |
| wavelet-LHH_firstorder_Median | 0.78 |
| wavelet-LLH_glszm_SizeZoneNonUniformity | 0.81 |
| original_glszm_SizeZoneNonUniformityNormalized | 0.83 |
| wavelet-LLH_gldm_GrayLevelNonUniformity | 0.88 |
| log-sigma-4-0-mm-3D_firstorder_Energy | 0.90 |
| log-sigma-4-0-mm-3D_firstorder_TotalEnergy | 0.78 |
| log-sigma-5-0-mm-3D_firstorder_TotalEnergy | 0.80 |
| wavelet-LLL_firstorder_10Percentile | 0.83 |
| wavelet-LLH_firstorder_Minimum | 0.79 |
| log-sigma-5-0-mm-3D_glcm_DifferenceVariance | 0.77 |
| wavelet-LLH_glszm_ZoneEntropy | 0.78 |
| log-sigma-2-0-mm-3D_glszm_GrayLevelNonUniformityNormalized | 0.77 |
| log-sigma-3-0-mm-3D_glcm_Imc1 | 0.91 |

Table S5. The selected features and corresponding ICC values of M_iTED-AP_ model.

| Feature | ICC |
| --- | --- |
| original_glrlm_RunVariance | 0.81 |
| original_gldm_DependenceNonUniformityNormalized | 0.80 |
| original_ngtdm_Coarseness | 0.78 |
| original_glszm_SizeZoneNonUniformityNormalized | 0.85 |

Table S6. The selected features and corresponding ICC values of M_iTED-PVP_ model.

| Feature | ICC |
| --- | --- |
| original_glcm_MaximumProbability | 0.84 |
| original_glrlm_LongRunLowGrayLevelEmphasis | 0.81 |
| original_glszm_LowGrayLevelZoneEmphasis | 0.82 |
| original_ngtdm_Coarseness | 0.86 |

Table S7. The selected features and corresponding ICC values of M_iTED-HBP_ model.

| Feature | ICC |
| --- | --- |
| original_firstorder_Uniformity | 0.80 |
| original_glcm_Id | 0.83 |
| original_glrlm_LongRunLowGrayLevelEmphasis | 0.79 |
| original_glrlm_LowGrayLevelRunEmphasis | 0.77 |
| original_glrlm_RunPercentage | 0.83 |
| original_glszm_LowGrayLevelZoneEmphasis | 0.84 |
| original_glszm_SmallAreaLowGrayLevelEmphasis | 0.90 |
| original_gldm_DependenceNonUniformityNormalized | 0.90 |
| original_ngtdm_Coarseness | 0.88 |


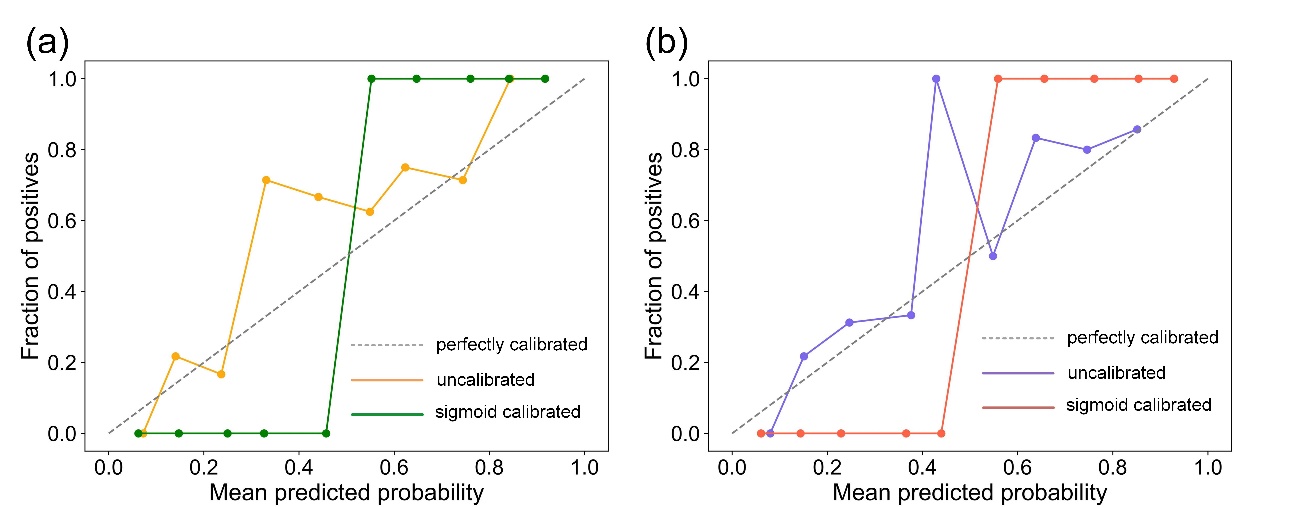


Figure S1. The model calibration curves of fusion_R model (a) and fusion_CR model (b) before and after calibration.
